# Improvement in The Function of Isolated Rat Pancreatic Islets through Reduction of Oxidative Stress Using Traditional Iranian Medicine

**Published:** 2014-05-25

**Authors:** Mahban Rahimifard, Mona Navaei-Nigjeh, Neda Mahroui, Sanaz Mirzaei, Zahra Siahpoosh, Pharm. D.4, Amir Nili-Ahmadabadi, Azadeh Mohammadirad, Maryam Baeeri, Reza Hajiaghaie, Mohammad Abdollahi

**Affiliations:** 1 Faculty of Science and Agriculture, Payame Noor University of Tehran, Tehran, Iran; 2 Faculty of Pharmacy and Pharmaceutical Sciences Research Centre, Tehran University of Medical Sciences, Tehran, Iran; 3 Department of Tissue Engineering, Faculty of Advanced Technologies in Medicine, Tehran University of Medical Sciences, Tehran, Iran; 4 Pharmaceutical Branch, Islamic Azad University of Medical Sciences, Tehran, Iran; 5 Pharmacognosy and Pharmaceutics Department of Medicinal Plants Research Center, Institute of Medicinal Plants, ACECR, Karaj, Iran

**Keywords:** Islets of Langerhans, Oxidative Stress, Medicine, Iranian Traditional, In Vitro

## Abstract

**Objective:**

Pancreatic islets have fewer antioxidant enzymes than other tissues and thus are vulnerable to oxidative stress. In the present study, the effects of nine specifically selected Iranian medical plants on the mitochondria function and survival of isolated rat islets were examined.

**Materials and Methods:**

In this experimental study, following laparotomy, pancreases of rats were removed and the islets isolated and incubated *in vitro* for 24 hours. Logarithmic doses of plant materials were added to the islets and incubated for an additional 24 hours after which the viability of the cells and production of reactive oxygen species (ROS) were measured. Levels of insulin production in relation to static and stimulated glucose concen- trations were also determined.

**Results:**

The tested compounds markedly increased survival of the islet cells, their mi- tochondrial activity, and insulin levels at the same time as reducing production of ROS. Greatest effects were observed in the following order: Peganum harmala, Glycyrrhiza glabra, Satureja hortensis, Rosmarinus officinalis, Teucrium scordium, Aloe vera, Zingiber officinale, Silybum marianum, and Hypericum perforatum at doses of 10, 10^3^, 10^4^, 10, 10^2^, 10^2^, 10^-1^, 10 and 10^3^μgmL^-1^, respectively.

**Conclusion:**

Based on these results, we suggest that pretreatment with these select- ed Iranian medical plants can improve the outcomes of pancreas transplants and grafts through the control of oxidative stress damage.

## Introduction

Diabetes mellitus (DM), which affects many organs in the body, is one of the most prevalent metabolic disorders in all parts of the world. Insulin and oral natural or synthetic hypoglycemic agents are used to treat DM but these drugs treat rather than cure the disease and have noticeable side effects (1).

Cell-based therapy for DM using activated pancreatic islet cells is theoretically possible as a cure for DM patients who don’t respond to current drugs (2, 3). A major drawback of this technique is that pancreatic islet cells face excessive oxidative stress during the isolation and transplantation procedures (4).

Oxidative stress is the result of an imbalance between antioxidants and free radicals involved in cellular damage (5). For instance, increasing reactive oxygen species (ROS) in pancreatic beta cells reduces insulin gene expression and insulin release and also damages the islets (6).

Nowadays, several plants are known for their antioxidant properties (7). Lessening oxidative stress through the use of herbal sources may improve survival of isolated rat pancreatic islets by scavenging harmful free radicals (3). Use of herbal medicine is not new. Herbal extracts have been used traditionally to cure diseases in every culture over the last 1000 years (8) and scientists today continue to investigate their potential to provide reliable therapies (9).

The present study aimed to screen the potential of selected herbs with strong anti-oxidative stress properties in relation to the viability and functionality of rat pancreatic islets. These herbs were identified from Persian folk medicine as listed in table 1.

## Materials and Methods

### Chemicals

All chemicals in this study were supplied from Sigma-Aldrich Co. (Gmbh, Munich, Germany). Rat specific insulin ELISA kit was obtained from Mercodia (Sweden). All other chemicals which were used in the study were obtained from standard commercial suppliers.

### Animals

Male adult Wistar rats (2-3 months), with a weight of approximately ~250 ± 25 g, from the animal house of the Faculty of Pharmacy, Tehran University of Medical Science (TUMS) were used. All animals were treated according to guidelines, which are approved by the TUMS Ethics Committee under the code number 90- 03-33-15668.

### Experimental protocols for islet isolation and culture

After the accommodation of the rats to the lab environment, the pancreas was removed and washed with Krebs buffer (8 NACL, 0.27 KCL, 0.42 NaH_2_PO_4_, 0.05 MgCL_2_, 2.38 HEPES, 0.22 CaCL_2_.2H_2_O, 0.5 glucose. 1H_2_O, in grams per liter, at pH=7.4) in order to remove lymph nodes, fat and blood vessels. The pancreas was then cut into small pieces on ice and washed two times by centrifuging at 3000 g for 60 seconds. Collagenase enzyme was added to the falcon tissue culture dishes to digest tissues surrounding the islets at 37˚C. Digestion was stopped by adding bovine serum albumin (BSA) and the pieces were again centrifuged twice at 3000 g for 60 seconds.

Islets approximately 100-150 μm in size were isolated by use of a sampler under a stereomicroscope. Standard RPMI 1640 culture medium containing fetal bovine serum (FBS), penicillin streptomycin (Pen Strep) and 8.3 mmol/l glucose was added to the fresh islets. They were then kept for 24 hours at 37˚C with 0.5% CO_2_ for experimental use (4).

### Plant materials

Nine medicinal herbs were obtained from the Research Institute of Medicinal Plants- ACECR in June 2009 and air-dried at room temperature. Samples were authenticated by a botanist (Y. Ajani), and verified specimens were preserved in the central herbarium of medicinal plants (ACECR). The scientific names and tested parts of the plant materials are detailed in table 2.

**Table 1:** List of Iranian medicinal plants and their usages


S. No.	Scientific name	Family	Antioxidant properties or medicinal use	Active compounds	Main component	References

**1**	Aloe vera	Liliaceae	Gastrointestinal disorders including peptic ulcer, antibacterial, anticancer, antifungal, antioxidant, anti-inflammatory and improves wound healing in diabetes	Phenolic compounds,glycosides(aloins), 8-C-ß-D-(2-O-(E)-coumaroyl)glucopyranosyl-2-(2-hydroxy)-propyl-7-methoxy-5-methylchromoone,acemannan,1,8-dihydroxyanthraquinone derivatives,β-1,4 acetylated mannan, mannosephosphate,alprogenglucoprotein, inorganiccompounds as Zn, Cr, Se, Mn, vitamins (A(ß-carotene), C and E)	Aloin (C_21_H_22_O_9_) 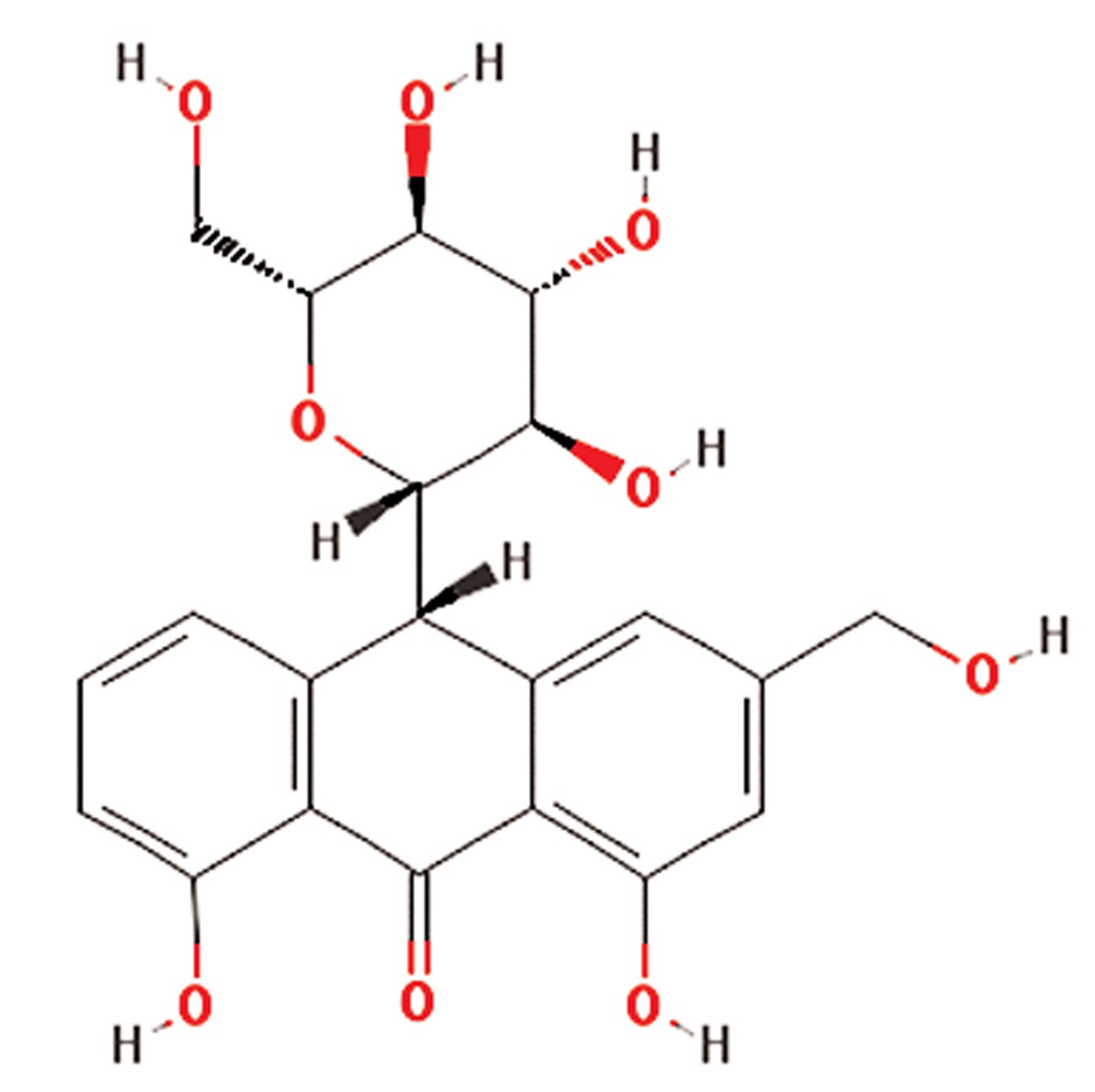	(10, 11)
**2**	Glycyrrhiza glabra	Fabaceae	Antioxidative, anti-inflammatory, anti-viral, treatment of fever, diarrhea and rheumatism	Glycyrrhizin, glycyrrhizinic acid, glabridin,glabrene, glabrol, licoflavonol, glycyrol, licoricone,formononetin, phaseollinisoflavan,hispaglabridin A&B, 3-hydroxy glabrol,3-methoxy glabridin, glabranin isomer,narigenin, and lupiwightenone	Glycyrrhizin (C_42_H_62_O_16_) 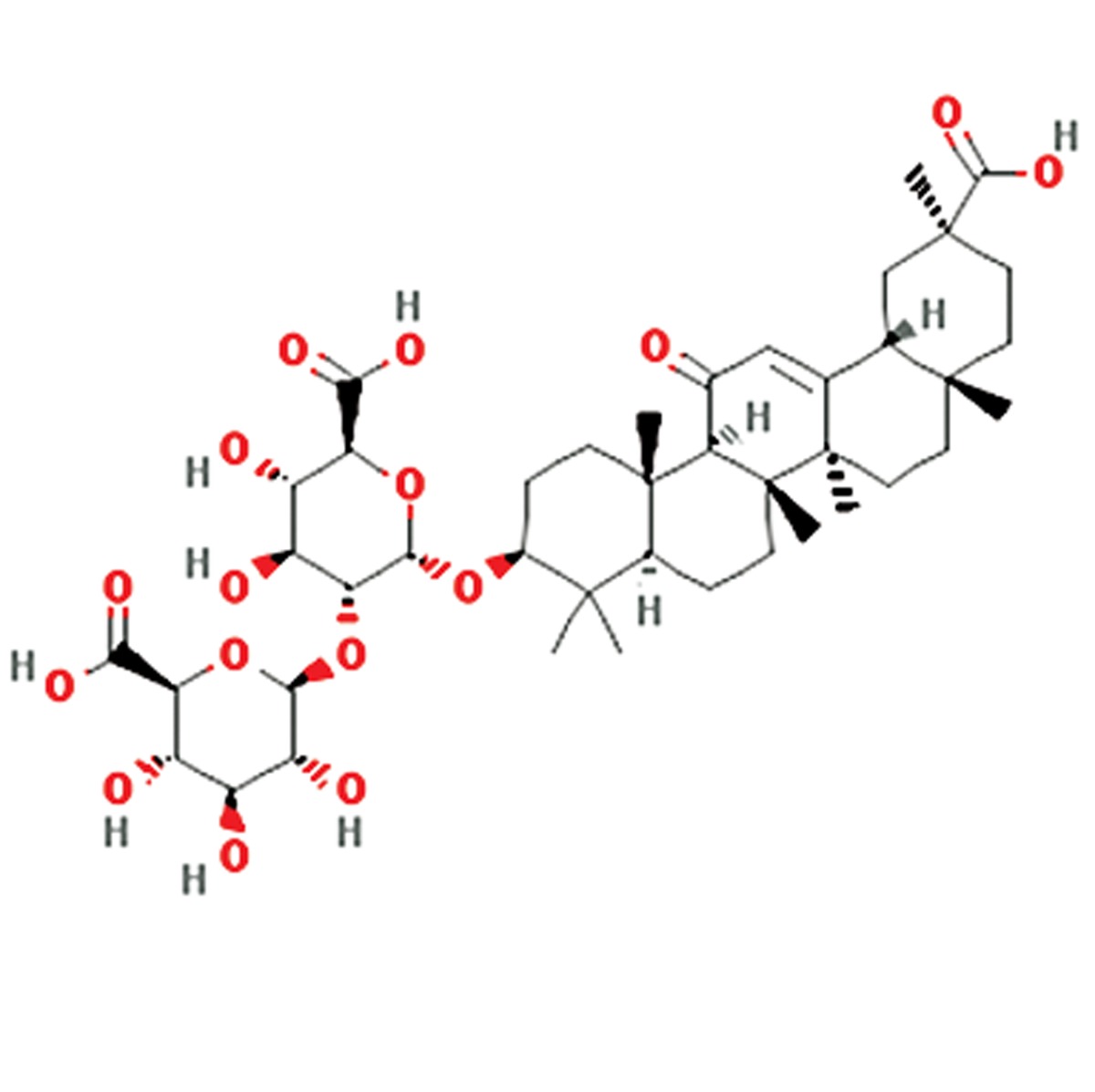	(6, 12)
**3**	Hypericum perforatum	Hypericaceae	Antibiotic, antiviral, antioxidant, anti-stress, anticancer, anti-depression	Hyperforin, flavonoids, phloroglucinols,naphthodiathrones, hypericin, hyperoside,quercetin, isoquercetin, quercitrin, rutin,campherol, luteolin, 13-118-biapigenin,1,3,6,7-tetra-hydroxyxanthone andprocyanidines	Hyperforin (C_35_H_52_O_4_) 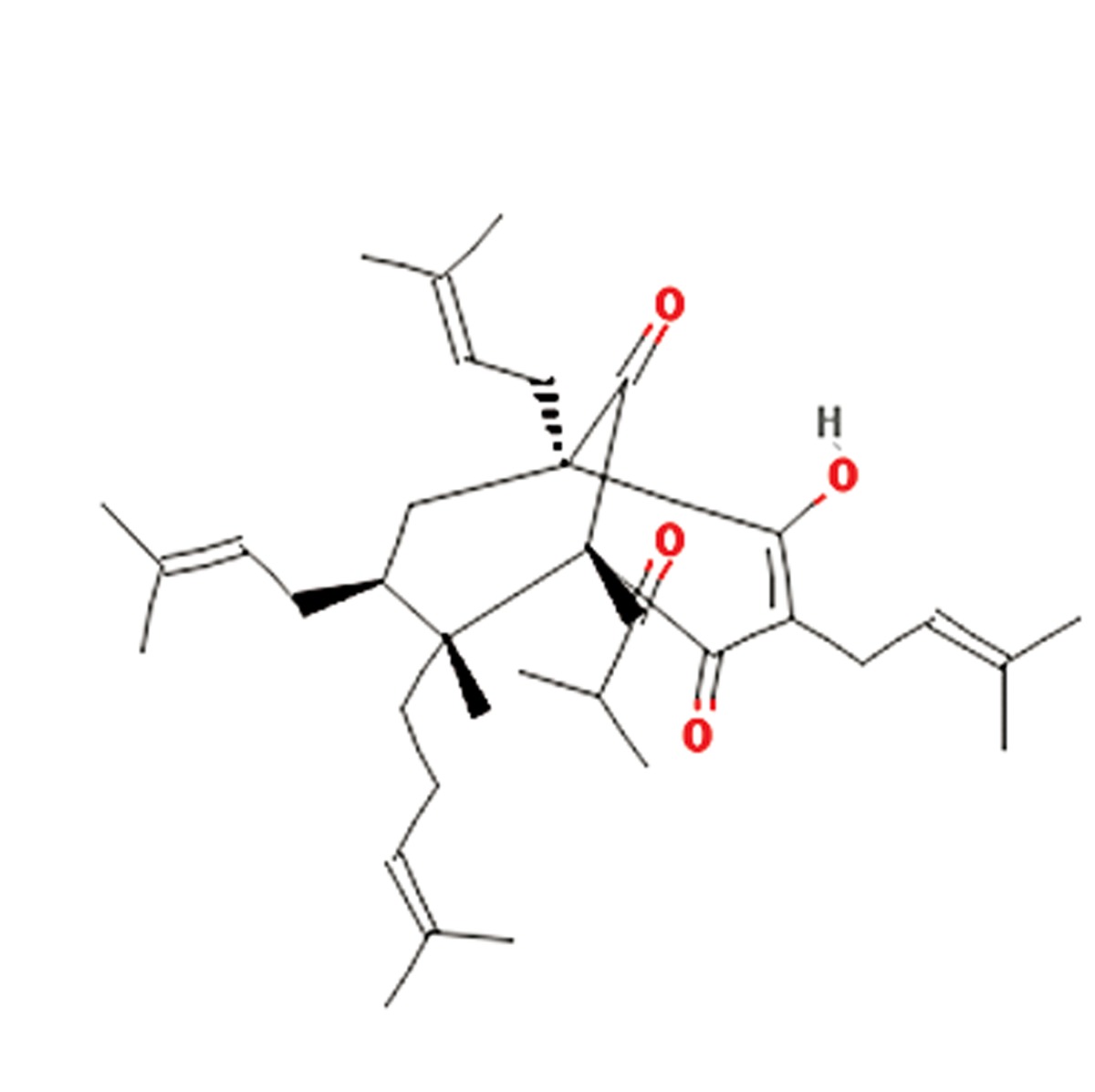	(13)
**4**	Peganum harmala	Nitrariaceae	Antibacterial, antiviral, antifungal, anticancer, antihistaminic, hypoglycemic effects	β-Carboline alkaloids such as harmaline,harmine, harmalol, harman, tetrahydroharmine,quinazoline derivatives, vasicine,vasicinon, anthroquinons and fixed oils	Harmaline (C_13_H_14_N_2O_) 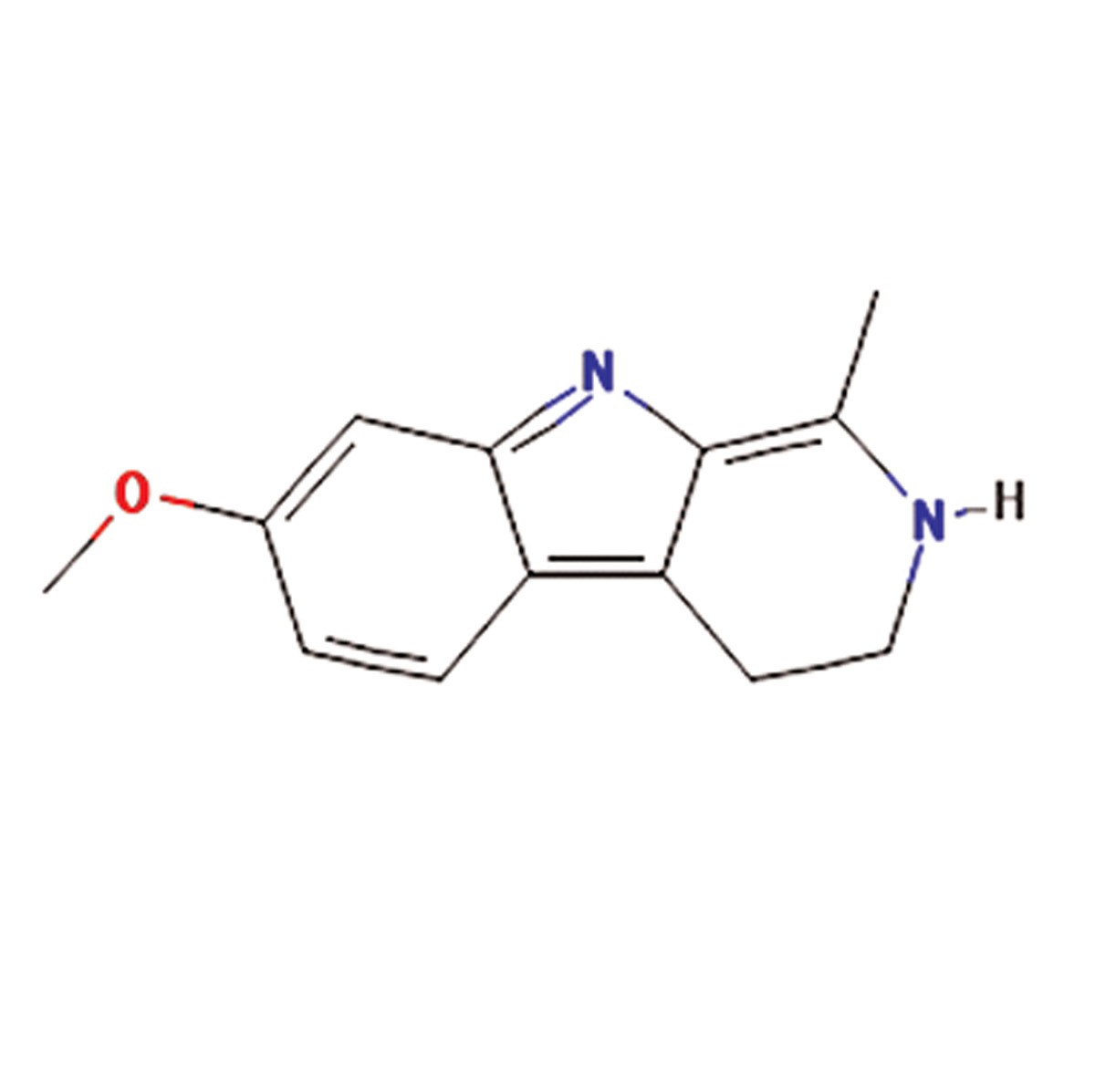	(14, 15)
**5**	Rosmarinus officinalis	Lamiaceae	Relaxing smooth muscles, hepatoprotective, anti-inflammatory, anti tumergenic activity	Polyphenolics, including rosemarinicacid, carnosic acid, carnosol, ursolic acid,abietane-type diterpenes, caffeic acid, flavonoides,triterpenes, and phenolic acids	Rosemaric acid (C_18_H_16_O_8_) 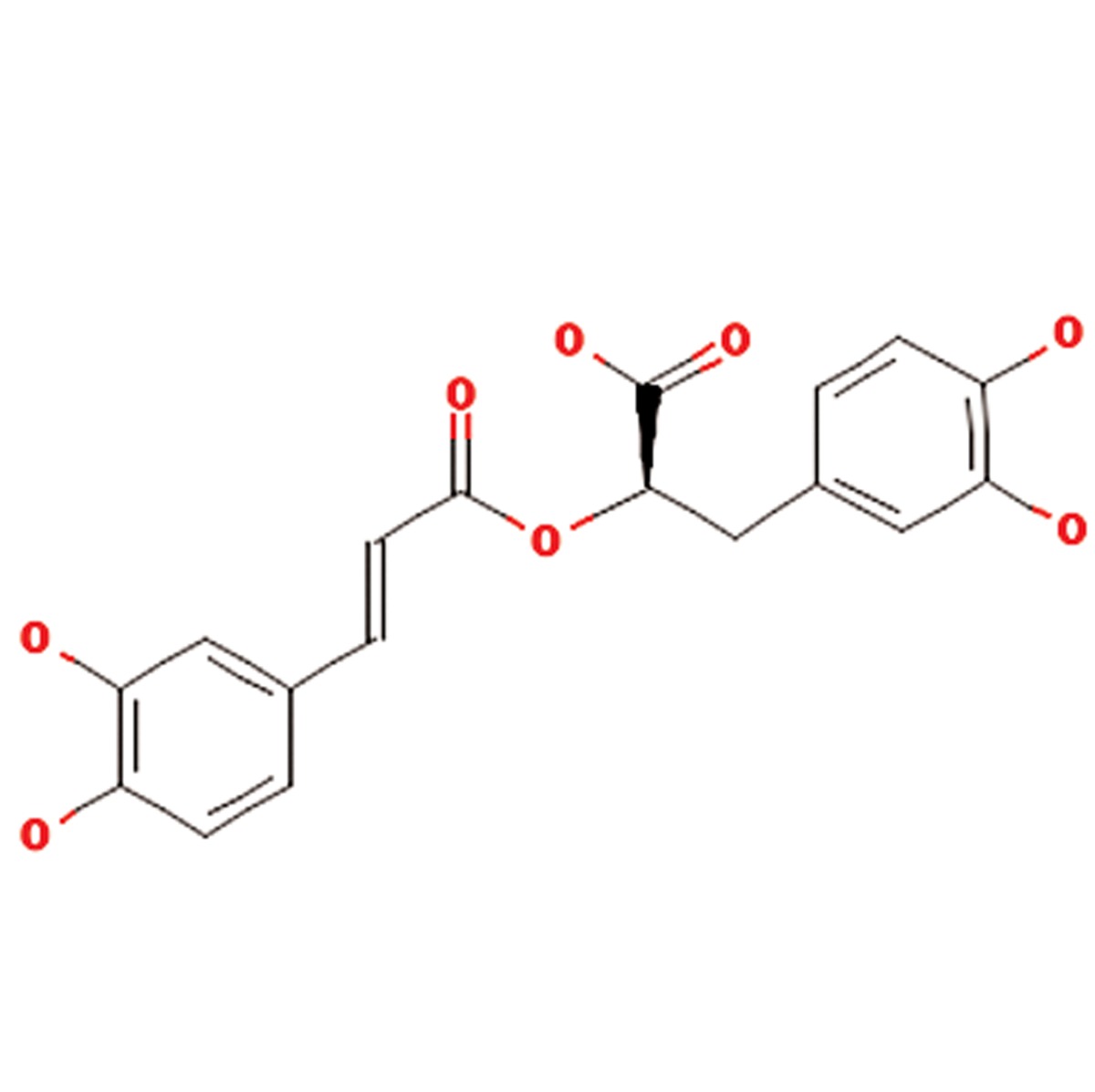	(7)
**6**	Satureja hortensis	Lamiaceae	Anti-diarrheal, antispasmodic, anti-inflammatory, antioxidant, antibacterial, antifungal, muscle pains, nausea, indigestion, diarrhea and infectious diseases	Carvacrol, thymol, , γ-terpinene, p-cymeneand linalool	Carvacrol (C_10_H_14_O)) 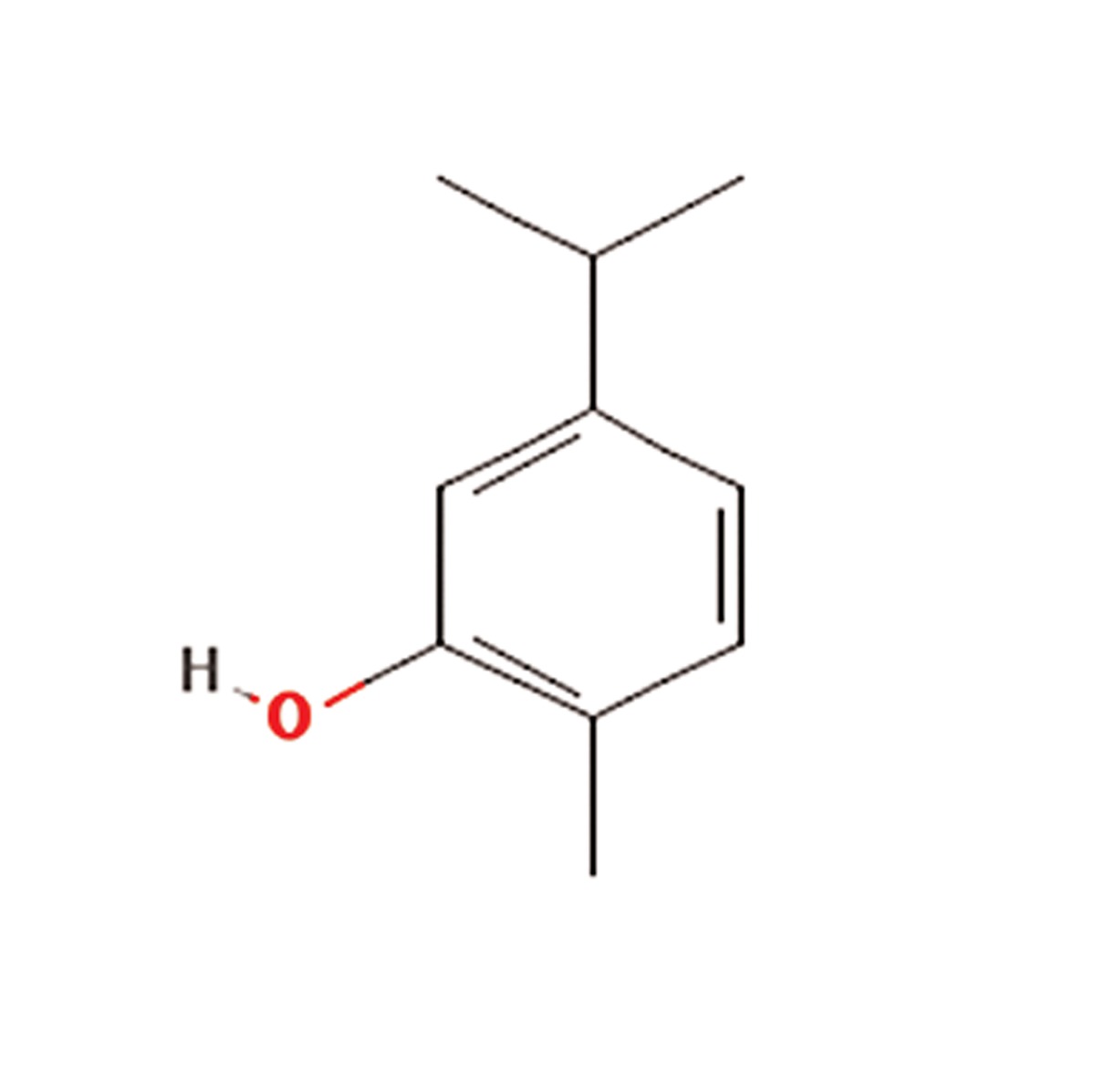	(16, 17)
**7**	Silybum marianum	Asteraceae	Silymarin or silybin (C_25_H_22_O_10_)	Silymarin flavonolignans (silibinin, alsocalled silybin, and its diastereoisomersisosilybin,silydianin, silychristin anddehydrosilybin), flavonoid taxifolin, betaine,trimethylglycine and essential fatty acids	Silymarin or silybin (C_25_H_22_O_10_ 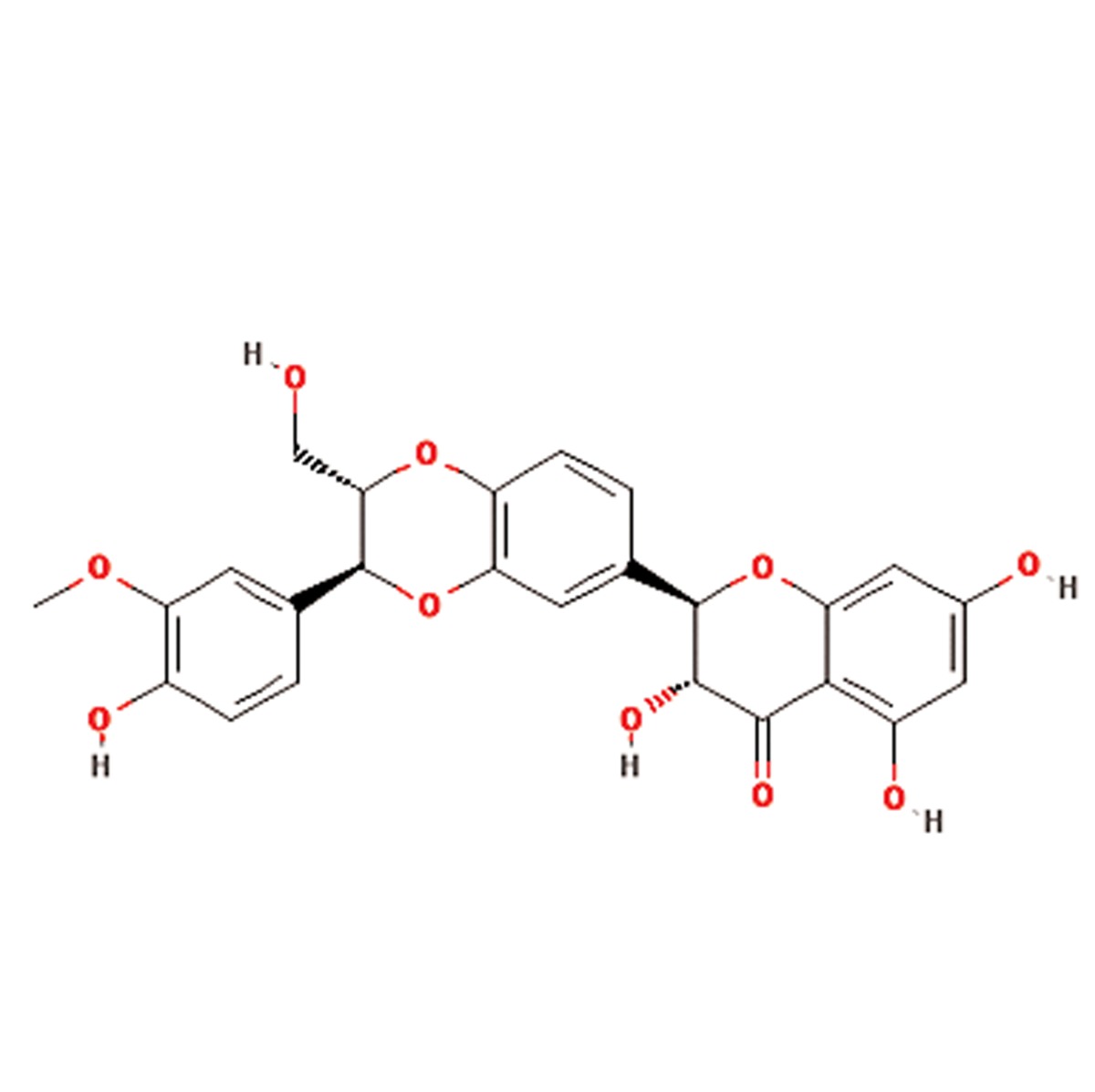	(18, 19)
**8**	Teucrium scordium	Lamiaceae	Antiinflammatory, antipyretic, antiseptic,astringent, diuretic, laxative, vermifuge,festering and inflamedwounds, bronchialailments, diarrhea, fever, hemorrhoids, andintestinal parasites	β-Caryophyllene, 6-AcetylteucjaponinB, (E)-b-farnesene, caryophyllene oxide,alpha-pinene and beta-pinene, β-farnesene,menthofuran, 1,8 cineole, β-eudesmol,α-humulene, diterpenes, flavonoids,saponnins, furanoid, pulegone, tannins andvolatile oil	β-Caryophyllene (C_15_H_24_O) 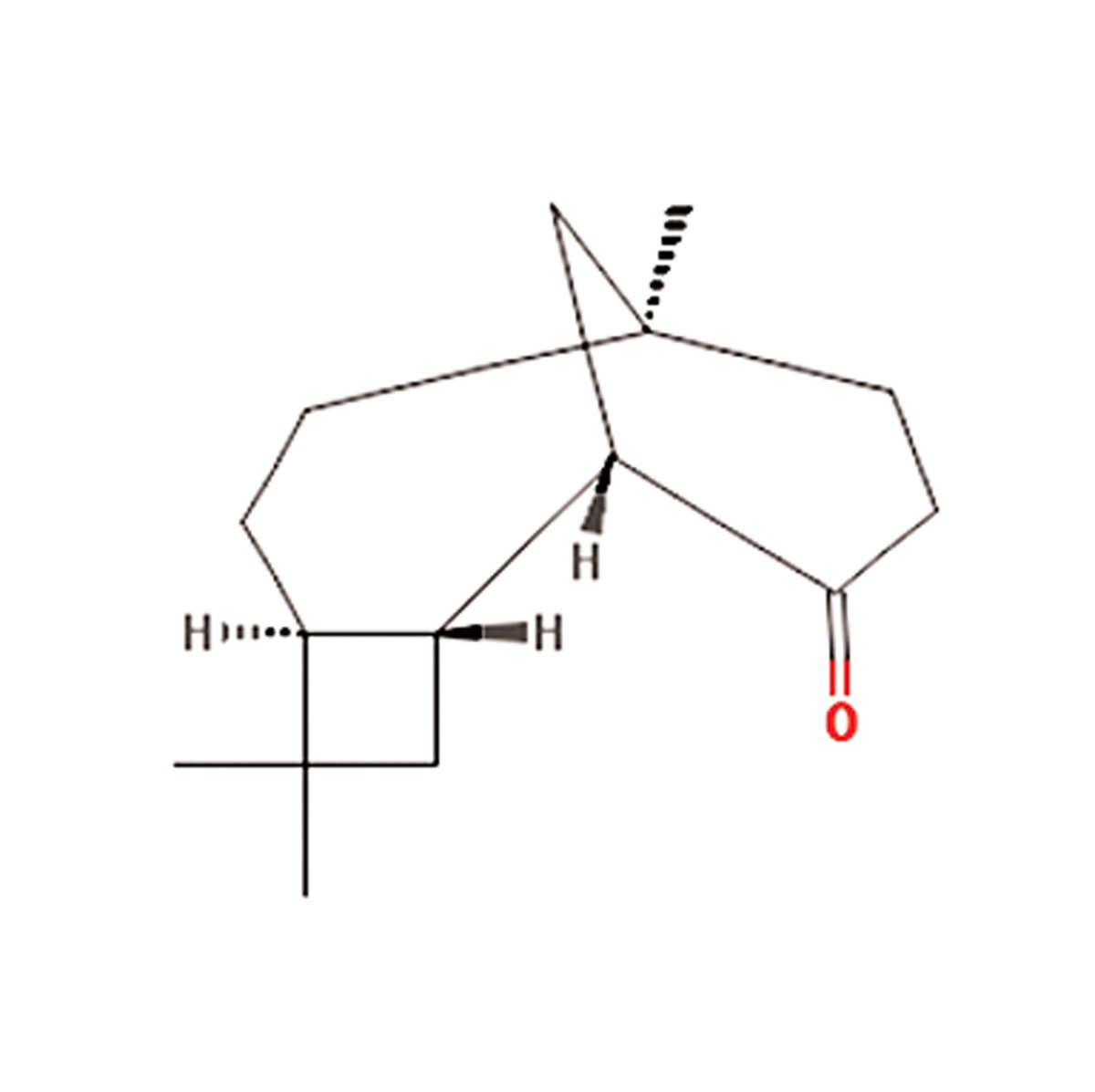	(20-22)
**9**	Zingiber officinale	Zingiberaceae	Antiinflammatory, antitumor, antilipedimic,antioxidant pharmacologic effects,Inhibited the induced hyperglycemia	Gingerols, zingerone, zingiberofficinalediol,zingibrene, shogaols, paradol and vanilloids	Gingerol (C_17_H_26_O_4_) 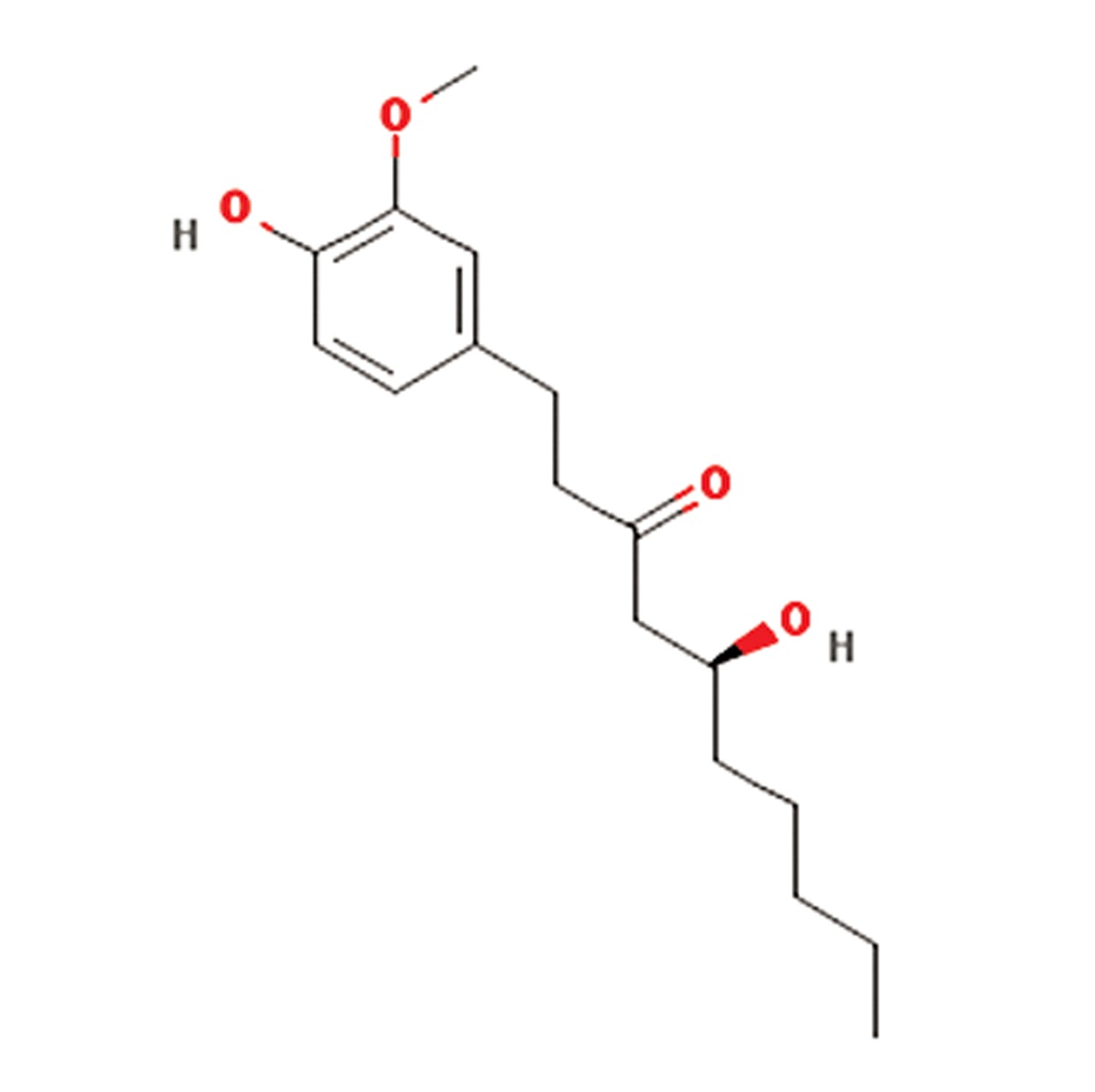	(23)


**Table 2:** Details of plant extractions.


S. No	Scientific name	Plant part used	Extraction yield (mgg^-1^)	Doses (µgmL^-1^)

**1**	Aloe vera	Gel	4.87	10, 10^2^, 10^3^, 10^4^
**2**	Glycyrrhiza glabra	Root	129.52	1, 10, 10^2^, 10^3^
**3**	Hypericum perforatum	Aerial parts	100.58	1, 10, 102, 10^3^
**4**	Peganum harmala	Seed	169.25	10^-1^, 1, 10, 10^2^
**5**	Rosmarinus officinalis	Aerial parts	236.51	10^-1^, 1, 10, 10^2^
**6**	Satureja hortensis	Aerial parts	134	10, 10^2^, 10^3^, 10^4^
**7**	Silybum marianum	Seed	123.49	1, 10, 10^2^, 10^3^
**8**	Teucrium scordium	Aerial parts	205	10, 10^2^, 10^3^, 10^4^
**9**	Zingiber officinale	Rhizome	140.57	10^-3^, 10^-2^, 10^-1^, 1


### Preparation of plant extracts and specific doses

Dried plant powder (40 g) was extracted by methanol using a percolation method at room temperature. Solvents were completely removed by drying under reduced pressure at 40˚C in a rotary evaporator. The samples were stored at 4˚C until used. Specifically for Aloe vera, the leaves (1000 g) were washed in a suitable bactericide (chlorhexidine). Sections were ground to a liquid, and the pulp was removed by filtering. The resultant gel was then freeze dried.

Various logarithmic concentrations from each plant extraction were made in RPMI medium culture and exposed to the islets for 24 hours at 37˚C. Doses of the plant materials are listed in table 2. To make sure of the range of doses, a pilot study was undertaken using doses from the literature.

### Measurement of metabolism in the islets

Mitochondrial metabolism in the islets was tested using dimethylthiazole-2-y1-2, 5-diphenyltetrazolium bromide (MTT) in order to investigate the effect of plant extracts on cell viability. After *in vitro* treatment with various doses of extracts for incubation periods of 24 hours, islets were washed twice with Krebs- HEPES and incubated with 20 μl of MTT (0.5 mg/mL) at 37˚C. This yellow solution was reduced and became purple in the living cells. After 3 hours, dimethyl sulfoxide (DMSO) was added and shaken for 30 minutes, then the absorbance of the samples was measured at 590 nm using a microplate reader (6).

### Measurement of insulin secretion in the islets

The level of insulin secretion in a glucose static enviroment was measured for investigating the function of the islets. Isolated islets which were cultured at 37˚C in a humidified atmosphere of 5% CO_2_ in air in RPMI-1640 medium were washed twice with Krebs-HEPES buffer. Wells which contained 10 islets were then incubated in 2.8 mM glucose in buffer for 30 minutes. After that, half of the islet groups were treated with 2.8 mM glucose (basal dose) and the others with 16.7 mM glucose (stimulant dose) for 30 minutes at 37˚C. At the end, insulin was measured in the medium using a rat insulin ELISA kit. Results are reported as insulin released in the medium.

### Measurements ROS production in the islets

2’, 7’-dichlorodihydrofluorescin diacetate (DCFHDA) was used to measure production of ROS and the oxidation of this compound was measured using an ELISA reader.

Each of the ten islets, which were collected in separate microtubes, was homogenized using extraction buffer and then centrifuged at 2375 g for 15 minutes. After that, 10 μl 2’,7’-dichlorodihydrofluorescin (DCFH) and 162 μl assay buffer was mixed together and 50 μl supernatant of the islet extraction was added. These solutions were incubated at 37˚C for 15 minutes. At the end, the absorbance of samples was measured every 10 minutes up to 60 minutes using a microplate reader. Remaining islets were collected for measurement of protein concentration, which was used to normalize the concentrations.

### Protein assay

Total protein level was measured by adding Bradford reagent dye to diluted samples and using BSA as the standard. After 5 minutes, the absorbance was read at 595 nm using a microplate reader.

### Statistical analysis

Data were expressed as the mean ± SEM of 3 different experiments, each read in duplicate. Oneway ANOVA followed by Tukey tests were used to analyze the data. Differences between groups were considered significant if the p value was lower or equal to 0.05. StatsDirect version 2.7.9 was used to analyze data.

## Results

### Peganum harmala (PH)

**Fig 1:**
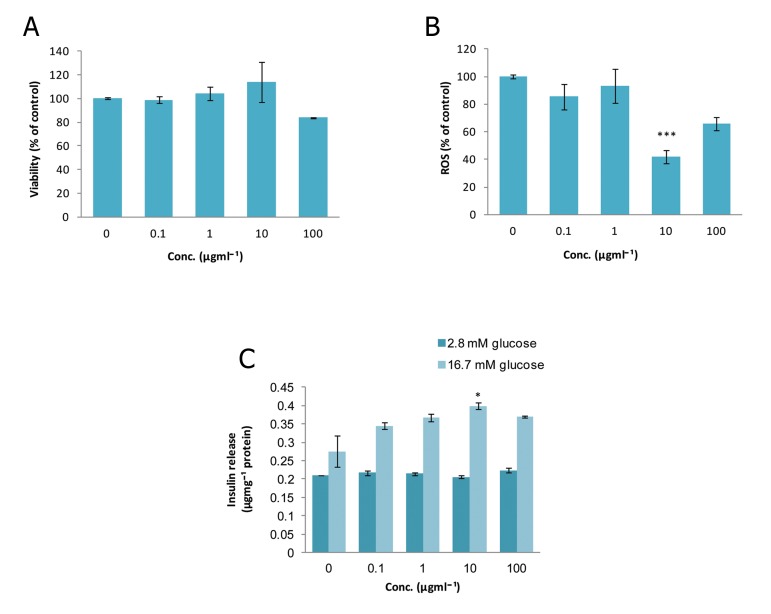
Effect of different concentrations of Peganum harmala on A. viability, B. level of ROS and C. released insulin from isolated rat pancreatic islets. Data are expressed as Mean ± SEM of 3 different experiments performed in duplicate. Significantly different from control group at ***; p<0.001 and *; p<0.05.

As shown in figure 1A, there was no significant change in the viability of islets at any concentration of PH compared to the control group. However, at a dose of 10 μgmL^-1^, a remarkable reduction (p<0.001) was observed in ROS production which decreased to 58% of that of the control (Fig 1B). Also, this concentration caused a significant increase (p<0.05) in insulin release in comparison to control group in the stimulated condition (16.7 mM glucose).

### Satureja hortensis (SH)

**Fig 2:**
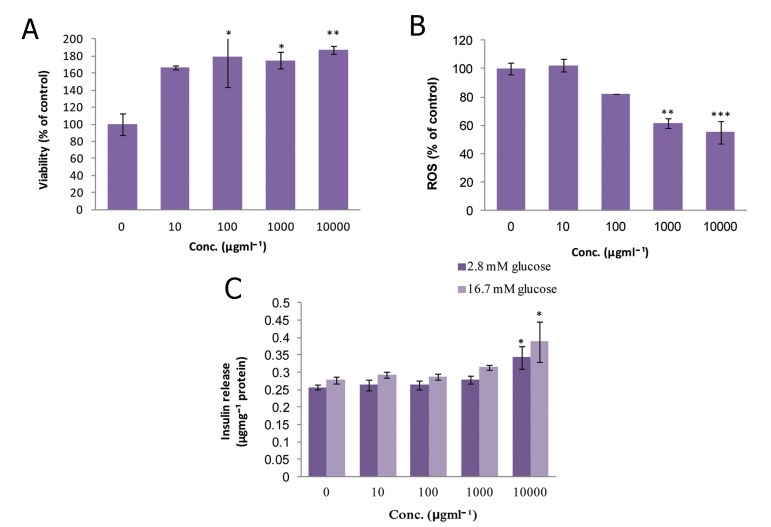
Effect of different concentrations of Satureja hortensison on A. viability, B. level of ROS and C. released insulin from isolated rat pancreatic islets. Data are expressed as Mean ± SEM of 3 different experiments performed in duplicate. Significantly different from control group at ***; p<0.001, **; p<0.01 and *; p<0.05.

The viability of islets at doses of 10^2^, 10^3^, and 10^4^ μgmL^-1^ of SH were considerably increased (p<0.05, p<0.05, and p<0.01 respectively) in comparison to the control group. Only the 10 μgmL^-1^ dose had no significant effect on cell viability (Fig 2A). As indicated in figure 2B, ROS production dropped significantly to 38.49% and 44.85% of that of the control group on exposure to doses of 10^3^ and 10^4^ μgmL^-1^ of SH (p<0.01 and p<0.001, respectively). Administration of SH at a concentration of 10^4^ μgmL^-1^ significantly increased (p<0.05) insulin secretion in the static (2.8 mM glucose) and stimulated (16.7 mM glucose) environment in comparison to the matched controls (Fig 2C).

### Rosmarinus officinalis (RO)

**Fig 3:**
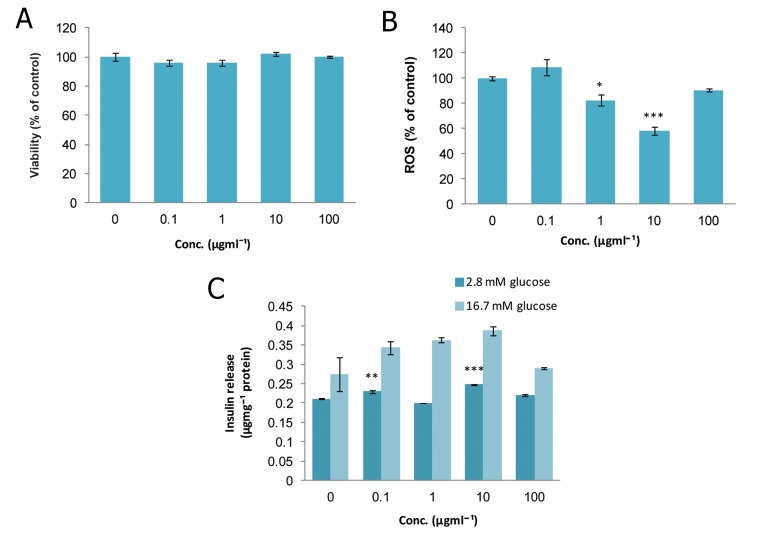
Effect of different concentrations of Rosmarinus officinalis on A. viability, B. level of ROS and C. released insulin from isolated rat pancreatic islets. Data are expressed as Mean ± SEM of 3 different experiments performed in duplicate. Significantly different from control group at ***; p<0.001, **; p<0.01 and *; p<0.05.

As shown in figure 3A, there was no significant change in the viability of islets at any concentration of RO compared to the control group. Results in figure 3B show that ROS production dropped significantly to 17.7% and 42% of that of the controls on exposure to doses of 1 and 10 μgmL^-1^ (p<0.05 and p<0.001, respectively). As indicated in Fig 3C, treatment of incubated islets in the basal state (2.8 mM glucose) with doses of 0.1 and 10 μgmL^-1^ meaningfully increased insulin secretion (p<0.01 and p<0.001, respectively), whereas in the stimulated state (16.7 mM glucose), only treatment with a dose of 10 μgmL^-1^ resulted in a significant relative increase in insulin secretion (p<0.05).

### Zingiber officinale (ZO)

**Fig 4:**
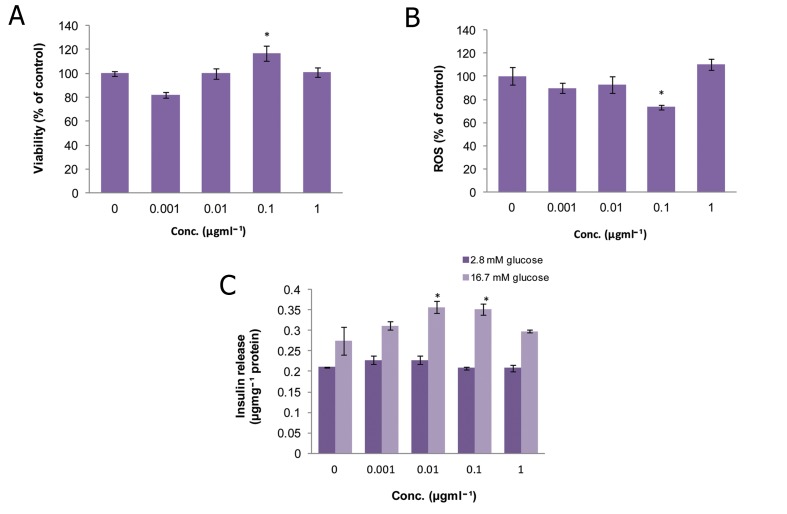
Effect of different concentrations of Zingiber officinale on A. viability, B. level of ROS and C. released insulin from isolated rat pancreatic islets. Data are expressed as Mean ± SEM of 3 different experiments performed in duplicate. Significantly different from control group at *; p<0.05.

As seen in figure 4A, the metabolic activity of islets in 10^-3^, 10^-2^, and 1 μgmL^-1^ of ZO did not significantly change but at 10^-1^ μgmL^-1^, the viability increased significantly (p<0.05) when compared with controls. Additionally, this concentration caused a significant decrease (p<0.05) in ROS production compared to the control group (Fig 4B) and reduced ROS to 26.6% of that seen in the controls. Administration of ZO at concentrations of 10^-2^ and 10^-1^ μgmL^-1^ significantly increased (p<0.05) the release of insulin in the stimulated state (16.7 mM glucose). However, there was no significant change in the secretion of insulin in the basal state (2.8 mM glucose) when compared to the matched control (Fig 4C).

### Sylibum marianum (SM)

**Fig 5:**
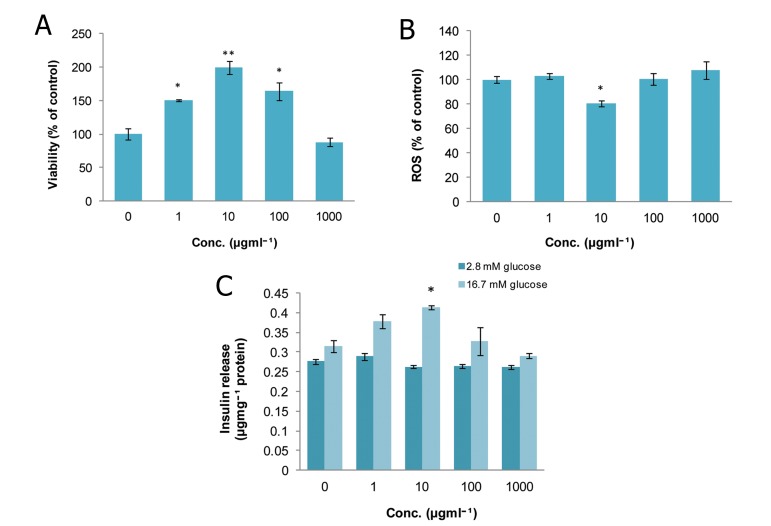
Effect of different concentrations of Sylibum marianum on A. viability, B. level of ROS and C. released insulin from isolated rat pancreatic islets. Data are expressed as Mean ± SEM of 3 different experiments performed in duplicate. Significantly different from control group at **; p<0.01 and *; p<0.05.

As seen in figure 5A, SM significantly increased the viability of the islets at concentrations of 1, 10 and 10^2^ μgmL^-1^, with the maximum change observed at 10 μgmL^-1^. This concentration also resulted in a significantly greater release of insulin (p<0.05) in comparison to the control group in the stimulated phase (16.7 mM glucose), and decreased ROS production to 19.25% of that seen in the control (Fig 5B).

### Aloe vera (AV)

**Fig 6:**
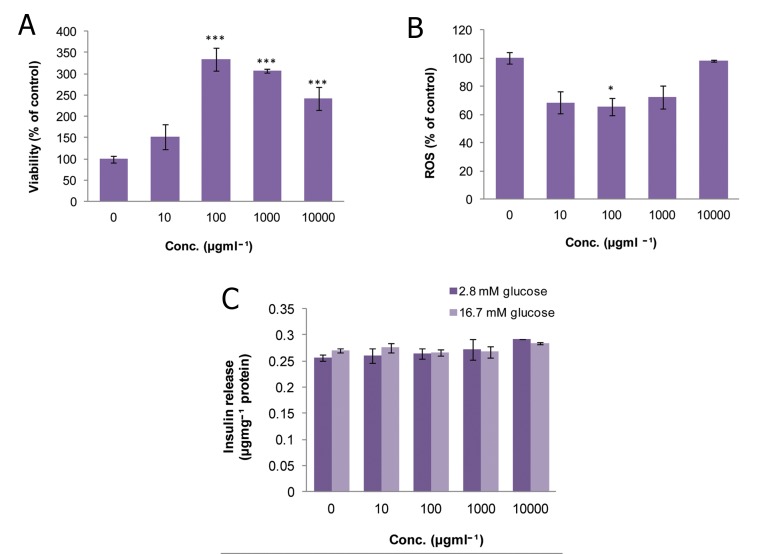
Effect of different concentrations of Aloe vera on A. viability, B. level of ROS and C. released insulin from isolated rat pancreatic islets. Data are expressed as Mean ± SEM of 3 different experiments performed in duplicate. Significantly different from control group at ***; p<0.001 and *; p<0.05.

The viability of islets at AV doses of 10^2^, 10^3^ and 10^4^ μgmL^-1^ was significantly higher (p<0.001) than in the control group. Only the dose of 10 μg mL^-1^ showed no significant effect on cell viability (Fig 6A). As indicated in figure 6B, there was no significant change in ROS production at doses of 10, 10^3^ and 10^4^ μgmL^-1^ but at a dose of 10^2^ μgmL^-1^ a significant reduction (p<0.05) was observed and ROS production decreased to 34.28% of that of the control. Results in figure 6C also show that there was no significant change in insulin release at any concentration of AV when compared with the matched control.

### Hypericum perforatum (HP)

**Fig 7:**
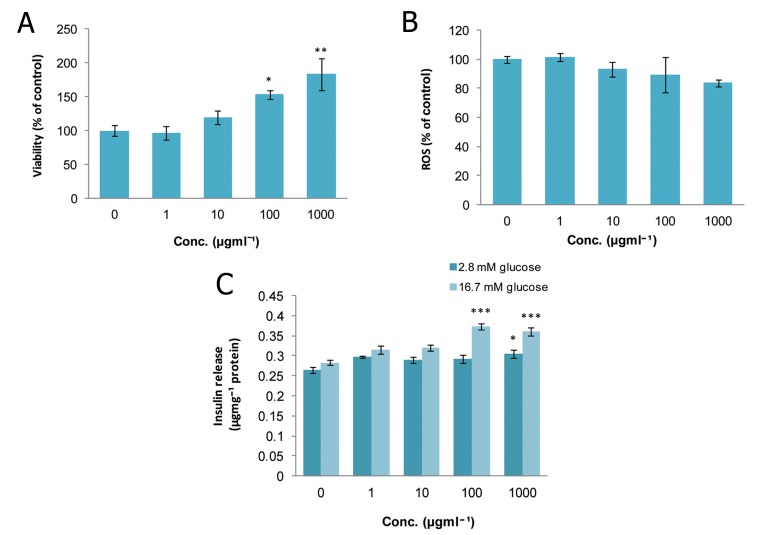
Effect of different concentrations of Hypericum perforatum on A. viability, B. level of ROS and C. released insulin from isolated rat pancreatic islets. Data are expressed as Mean ± SEM of 3 different experiments performed in duplicate. Significantly different from control group at ***; p<0.001, **; p<0.01 and *; p<0.05.

As seen in figure 7A, HP significantly increased the metabolic activity of islets at doses of 10^2^ and 10^3^ μgmL^-1^. Despite the lack of significant differences in viability of islets at doses of 1 and 10 μgmL^-1^, the observed changes in the concentrations studied were dose-dependent. Results in figure 7B showed that ROS production in the islets did not significantly change in comparison to the control group. As indicated in figure 7C, in the basal state (2.8 mM glucose) only treatment of the incubated islets with a dose of 10^3^ μgmL^-1^ HP increased insulin secretion significantly (p<0.05), whereas in the stimulated state (16.7 mM glucose) treatment of the incubated islets with doses of 10^2^ and 10^3^ μgmL^-1^ significantly increased insulin secretion (p<0.001).

### Teucrium scordium (TS)

**Fig 8:**
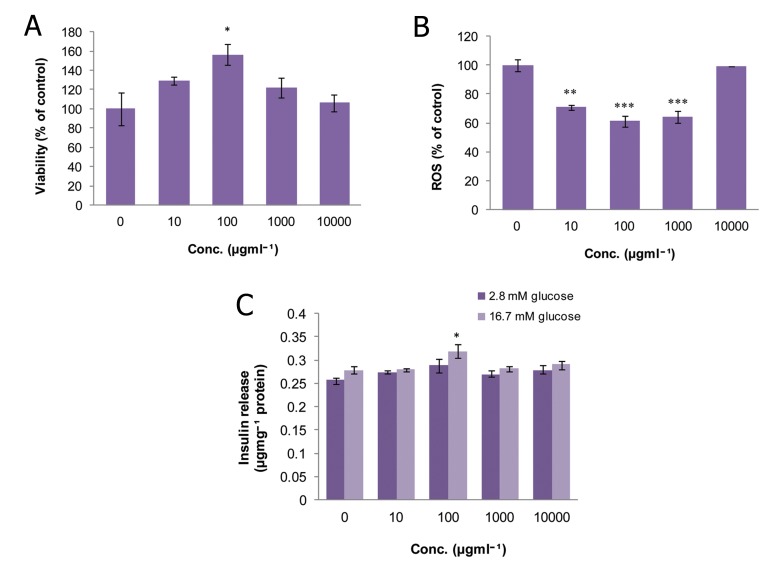
Effect of different concentrations of Teucrium scordium on A. viability, B. level of reactive oxygen species ROS and C. released insulin from isolated rat pancreatic islets. Data are expressed as Mean ± SEM of 3 different experiments performed in duplicate. Significantly different from control group at ***; p<0.001, **; p<0.01 and *; p<0.05.

As seen in figure 8A, the metabolic activity of islets at concentrations of 10, 10^3^, 10^4^ μgmL^-1^ TS did not change significantly, but at 10^2^ μgmL^-1^ the viability increased in comparison to the control group. In addition, this concentration caused a significant increase (p<0.05) in insulin release in comparison to the control group in the stimulated phase (16.7 mM glucose). However, administration of TS at other concentrations had no significant effect on insulin secretion (Fig 8C). TS also significantly decreased ROS production at concentrations of 10, 10^2^ and 10^3^ μgmL^-1^, with maximum change observed at 10^2^ μgmL^-1^. The dose of 10^2^ μgmL^-1^ decreased ROS production to 38.55% of that for the control (Fig 8B).

### Glycyrrhiza glabra (GG)

**Fig 9:**
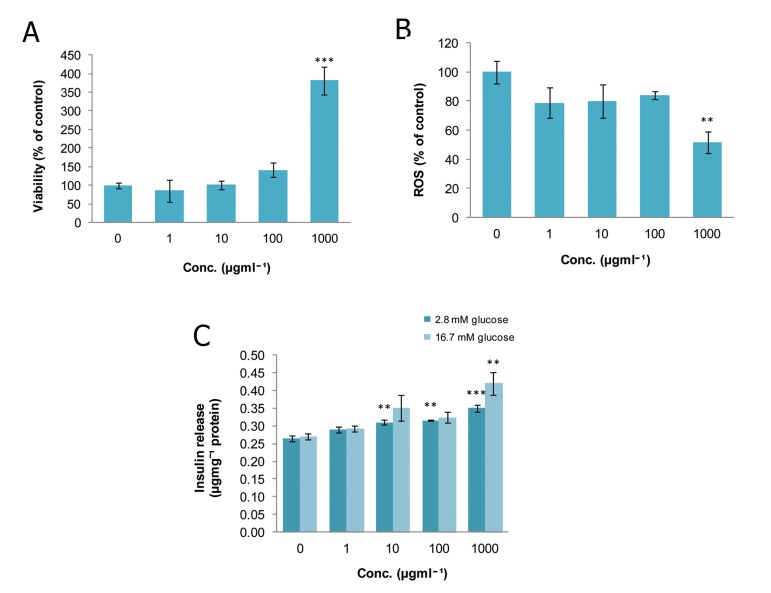
Effect of different concentrations of Glycyrrhiza glabra on A. viability, B. level of ROS and C. released insulin from isolated rat pancreatic islets. Data are expressed as Mean ± SEM of 3 different experiments performed in duplicate. Significantly different from control group at ***; p<0.001 and **; p<0.01.

As seen in figure 9A, the metabolic activity of islets in 1, 10, 10^2^ μgmL^-1^ GG did not significantly change but at 10^3^ μgmL^-1^, viability increased significantly (p<0.001). Additionally, this concentration resulted in a significant decrease (p<0.01) in ROS production (Fig 9B), reducing ROS to 48.5% of that of the control. As indicated in figure 9C, insulin secretion in the basal state was significantly increased at doses of 10, 10^2^, and 10^3^ μgmL^-1^ GG (p<0.01, p<0.01, and p<0.001, respectively). Only a concentration of 1 μgmL^-1^ was observed to have no significant effect on insulin secretion. Furthermore, administration of a 10^3^ μgmL^-1^ dose of GG significantly increased (p<0.01) insulin secretion in the stimulated (16.7 mM glucose) state when compared with the matched control.

## Discussion

Pancreatic islets have fewer antioxidant enzymes than other tissues and are thus more vulnerable to oxidative stress. Augmenting antioxidant protection mechanisms in pancreatic islets helps them to fight oxidative stress, especially during the implant process. The present findings showed a significant improvement in the antioxidant status of isolated islets exposed to extracts of several famed Iranian plants; PH, GG, SH, RO, TS, AV, ZO, SM, and HP. These plant extracts reduced ROS production to 58, 48.5, 44.85, 42, 38.55, 34.28, 26.6, 19.25, and 16.14% of that of the controls at their effective doses; 10 μgmL^-1^, 10^3^ μgmL^-1^, 10^4^ μgmL^-1^, 10 μgmL^-1^, 10^2^ μgmL^-1^, 10^2^ μgmL^-1^, 10^-1^ μgmL^-1^, 10 μgmL^-1^ and 10^3^ μgmL^-1^ respectively. Interestingly, not only a reduction in ROS production but also an increase in released insulin was observed with the use of all the extracts except Aloe vera. Moreover, at the selected doses used no trace of toxicity was seen for any of the plant extracts.

In this report, we have indicated that extract obtained from the seeds of PH is associated with the greatest improvement in the antioxidant defense of isolated pancreatic islets. PH decreased ROS to 58% of that for the controls in parallel with an increased release of insulin. The antioxidant activity of the principal compounds in PH has been indicated previously in numerous studies. Also, it has been reported that PH contains flavonoids. These are known to scavenge free radicals (24, 25); modulate the expression of genes encoding antioxidant enzymes, such as glutathione reductase, glutathione peroxidase, glutathione-S-reductase, and quinone reductase (26); and enhance the expression of other intracellular endogenous antioxidants, such as superoxide dismutase (SOD), catalase (CAT) and glutathione peroxidase (GPX) (27). In addition, Kim et al. (28) have investigated the protective effects of PH alkaloids against the damage induced by oxidative stress and showed that β-carbolines depress the loss of viability in PC- 12 cells through a scavenging action on ROS and inhibition of the oxidation of thiols. Furthermore, Berrougui et al. (24) have shown that alkaloids from the seeds of pH exert an antioxidant effect on the *in vitro* oxidation of low-density lipoproteins (LDLs) by decreasing conjugated diene and monoamine oxidase (MDA) formation. The active ingredient in this plant is mostly harmaline and its activity is thought to be mediated by chelating Cu^2+^, scavenging free radicals and thus inhibiting the oxidation of human LDLs. The change of β-carboline to dihydro-β-carboline (i.e. harmine to harmaline) seems to increase the potency of the antioxidant activity of the alkaloids (24). In the present study, we have observed that 10 μgmL^-1^ of pH is the effective dose in relation to oxidative stress and antioxidant status in isolated islets.

It has been observed that both the ethanolic extract and the essential oil of SH reverse the oxidative damage to rat lymphocytes induced by hydrogen peroxide (29). Another study has indicated that SH has a high phenolic and flavonoid content (128 mg gallic acid equivalents/g dried extract and 88.5 mg catechin equivalents/g dried extract, respectively). The results of this study clearly showed that SH has the ability to scavenge ABTS and DPPH radicals as well as reduce the ability of ferric ions in a dose-dependent manner due to the phenolic content of the extract. Additionally, this study suggests that SH is a natural, rich source of antioxidants for probable pharmaceutical application in the prevention of many free radical-associated diseases (30). Based on our data, it can be concluded that SH improves the antioxidant status of isolated islets in a dose-dependent manner and its effective dose decreases ROS to 44.85% as well as increasing insulin release.

Previous studies on RO have shown that the antioxidant activity of its extracts is mainly ascribable to phenolic compounds classified into three groups including phenolic diterpenes possessing an abietic acid framework, flavonoids, and phenolic acids (31, 32). The principle relevant constituents are composed of vast numbers of polyphenolics including carnosic acid, carnosol, rosemarinic acid, ursolic acid, etc. (33). Among these, carnosic acid and carnosol, abietane-type diterpenes, caffeic acid and its derivative, and rosmarinic acid are the main antioxidant compounds present in RO (34, 35). In one study, the diterpenes and genkwanin (a flavonoid component of RO) showed membrane-rigidifying effects, which may contribute to their antioxidant capacity through hindering diffusion of free radicals (36). In another study, it was demonstrated that aqueous extract of RO can be efficient against the oxidative stress secondary to experimental diabetes (37). In addition, due to its potent antioxidant properties, the RO extract exerts a remarkable antidiabetogenic effect (38). The antioxidant effects of RO shown here are in agreement with previous reports and our data show that the extract obtained from aerial parts of RO improves antioxidant status in isolated islets and produces its greatest effects, including significant enhancement of insulin release at 10 μg mL^-1^.

Previous work has shown that the active ingredients of ZO roots, such as zingerone, zingiberofficinalediol, bisabolene, gingerols, zingibrene, shogaols and paradol produce antioxidant activity (39). It has been shown to contain vitamin B, C and minerals such as calcium, magnesium, potassium, phosphorus and linoleic acid (40). Although data on ZO are very scant and depend mostly on *in vitro* data, it can be said that ZO has the potential to inhibit ROS in human erythrocytes (41), and prevent malathion-induced ROS-mediated toxicity through its curcumin and zingerone constituents (42). The 6-gingerol is another effective constituent of ZO that scavenges ROS (43, 44). In addition, other findings have shown that ginger extract reduces blood glucose in a dose dependent manner. Action may be via stimulation of insulin production from the pancreatic islets or by increasing peripheral utilization or inhibition of the proximal tubular reabsorption mechanism for glucose in the kidney (45). Our results are in agreement with the earlier reports indicating that ZO is a strong activator of insulin release and antioxidant in isolated islets at an effective dose of 10^-1^ μgmL^-1^.

SM is also a plant that contains lots of phenolic compounds such as flavonolignans, flavonoid taxifolin, and some other minor compounds. Silymarin, a standardized extract from the seeds of SM contains mainly silibinin and a minority of other phenolic compounds (46, 47). Silymarin or silybin acts through the scavenging of free radicals (48), modulations of antioxidant and inflammatory enzymes (49, 50), inhibition of mitogenic and cell survival signaling or modulations of cell cycle regulators (51). Silymarin is considered as one of the most effective drugs used in type II diabetes mellitus (52) and may be therapeutically beneficial for type I diabetes too (53). Moreover, it has been reported that silymarin causes significant reduction in blood free fatty acids and triglyceride (54). Previous findings showed that silymarin improves β-cell function in obese rats (55). In the present study, we tried to determine the effective dose of SM extract for treatment of isolated rat pancreatic islets. A concentration of 10 μgmL^-1^ not only decreased ROS to 19.25% of the level in controls, but also enhanced insulin release.

Aloe vera (Aloe barbadensis) is one of the medicinal plants, which is a traditionally well approved in the management of diabetes. Many studies report that the high contents of phenolic compounds, glycosides (aloins), 1, 8-dihydroxyanthraquinone derivatives, β-1, 4 acetylated mannan, mannosephosphate, alprogenglucoprotein, inorganic compounds such as Zn, Cr, Mn, vitamin E, and vitamin C in AV are responsible for its biological action (56, 57). Among these components, it has been shown that the total phenols, flavonoids, vitamin C and vitamin E in AV gel extract directly scavenge reactive oxidants; they are assumed to organize a vital endogenous defense against oxidative cell and tissue injury (58). In addition, the increased levels of plasma insulin observed in previous studies suggest that the antihyperglycemic activity of AV could be due to insulinogenic activity of the gel extract (59). Our results are in consonance with earlier reports of the antioxidant activity of AV as a significant reducer of ROS. Nevertheless, there was no change in insulin release from the isolated islets when they treated with our selected dose of AV (10-10^4^ μgmL^-1^).

Recently, it has been shown that HP extracts are highly effective in scavenging ROS and inhibiting ascorbate/iron-induced lipid peroxidation in rat cortical synaptosomes (60). HP has also been reported for its potent antihyperglycemic activity in diabetic rats. It seems that, HP stimulates insulin action and increases the pancreatic secretion of insulin (61). HP contains several phytochemical constituents such as rutin, flavonoids including quercetin and isoquercetin (62). For example, rutin has been reported to enhance insulin release and decrease blood glucose in diabetic animals (63). Our results are consistent with earlier reports of a marked increase in insulin secretion from isolated islets in a dose-dependent manner.

Previous findings have demonstrated the presence of diterpenes, flavonoids, saponnins, tannins and volatile oil in the aerial parts of TS. It has been suggested that this plant is a source of natural b-caryophyllene and (E)-b-farnesene and caryophyllene oxide (64-66). Additionally, other major compounds were β-farnesene, menthofuran, 1,8 cineole and α-humulene (67). Moreover, Kovacevic et al. (66) reported that TS oil was rich in alpha-pinene and beta-pinene (68). Although a survey of the literature produced no report on the antioxidant activity of this plant, our data showed that the extract obtained from aerial parts of TS improves antioxidant status in isolated islets and produces its greatest effects at a dose of 10^2^ μgmL^-1^; a dose which simultaneously enhanced insulin release.

The isolation of various chemical constituents from GG has been reported previously (Table 1). Among these constituents, the isoflavone derivatives of GG have a protective role against oxidative stress (68,69). In addition, liquorice polyphenol extract prevents ROS-induced injury to cells by permeating cell membranes and increasing intracellular antioxidant activity (70). On the other hand, previous studies have shown that glycyrrhizin is quite effective against hepatotoxicity, hyperglycemia, hyperlipidemia and associated oxidative stress, and thus a useful treatment for diabetes (71, 72). In addition, the results of a recent study indicated that glycyrrhizic acid may be a good candidate for islet transplantation procedures to maintain islets in a viable and functional state (6). In another study, Kalaiarasi and Pugalendi (73) have shown that glycyrrhetinic acid enhances plasma insulin and reduces the gluconeogenic enzymes in the liver and kidney of diabetic rats. Our results are in consistent with earlier reports and prove a marked insulin secretory effect in isolated islets in a dose-dependent manner. The effective dose of GG decreased ROS up to 48.5%.

## Conclusion

Taken together, the present findings show extracts from several Iranian plants; PH, GG, SH, RO, TS, AV, ZO, SM, and HP produce a significant improvement in the markers involved in the successful transplantation of islets, including viability of cells, ROS and insulin secretion. Based on these results, concentrations of 10 μgmL^-1^, 10^3^ μgmL^-1^, 10^4^ μgmL^-1^, 10 μgmL^-1^, 10^2^ μgmL^-1^, 10^2^ μgmL^-1^, 10^-1^ μgmL^-1^, 10 μgmL^-1^ and 10^3^ μgmL^-1^ are respectively the effective doses of these plants for isolated islets. Their effects on insulin release are especially promising in the treatment of diabetes. On the basis of these findings, the authors suggest that the next step is to test combinations of some of the most effective of these plants with the hope of producing a more efficient cell-based therapy for diabetes through the prevention of oxidative stress in isolated islets and improving mitochondrial function during the implant process. Although safety issues should not be forgotten, the efficacy of these extracts in the clinic should be studied according to guidelines to see whether these alone or in combination can control diabetes or maintain vitality of cells during the transplantation pancreatic islets. The present findings are very promising and pave the way to success in further studies.
